# Collective Motions and Mechanical Response of a Bulk of Single-Chain Nano-Particles Synthesized by Click-Chemistry

**DOI:** 10.3390/polym13010050

**Published:** 2020-12-25

**Authors:** Jon Maiz, Ester Verde-Sesto, Isabel Asenjo-Sanz, Peter Fouquet, Lionel Porcar, José A. Pomposo, Paula Malo de Molina, Arantxa Arbe, Juan Colmenero

**Affiliations:** 1 Centro de Física de Materiales (CFM) (CSIC-UPV/EHU)-Materials Physics Center (MPC), Paseo Manuel de Lardizábal 5, 20018 Donostia-San Sebastián, Spain; mariaester.verde@ehu.eus (E.V.-S.); misabel_asenjo@ehu.eus (I.A.-S.); josetxo.pomposo@ehu.eus (J.A.P.); paula_malo001@ehu.eus (P.M.d.M.); mariaaranzazu.arbe@ehu.eus (A.A.); juan.colmenero@ehu.eus (J.C.); 2 IKERBASQUE-Basque Foundation for Science, Plaza Euskadi 5, 48009 Bilbao, Spain; 3 Institut Laue-Langevin, 71 Avenue des Martyrs, CEDEX 9, 38042 Grenoble, France; fouquet@ill.fr (P.F.); porcar@ill.eu (L.P.); 4 Departamento de Polímeros y Materiales Avanzados: Física, Química y Tecnología, Universidad del País Vasco-Euskal Herriko Unibertsitatea (UPV/EHU), 20018 Donostia-San Sebastián, Spain; 5 Donostia International Physics Center, Paseo Manuel de Lardizábal 4, 20018 Donostia-San Sebastián, Spain

**Keywords:** single-chain nano-particles, polymer melts, intra-molecular cross-links, dynamic structure factor, rheology

## Abstract

We investigate the effect of intra-molecular cross-links on the properties of polymer bulks. To do this, we apply a combination of thermal, rheological, diffraction, and neutron spin echo experiments covering the inter-molecular as well as the intermediate length scales to melts of single-chain nano-particles (SCNPs) obtained through ‘click’ chemistry. The comparison with the results obtained in a bulk of the corresponding linear precursor chains (prior to intra-molecular reaction) and in a bulk of SCNPs obtained through azide photodecomposition process shows that internal cross-links do not influence the average inter-molecular distances in the melt, but have a profound impact at intermediate length scales. This manifests in the structure, through the emergence of heterogeneities at nanometric scale, and also in the dynamics, leading to a more complex relaxation behavior including processes that allow relaxation of the internal domains. The influence of the nature of the internal bonds is reflected in the structural relaxation that is slowed down if bulky cross-linking agents are used. We also found that any residual amount of cross-links is critical for the rheological behavior, which can vary from an almost entanglement-free polymer bulk to a gel. The presence of such inter-molecular cross-links additionally hinders the decay of density fluctuations at intermediate length scales.

## 1. Introduction

A new field starts emerging during the last years on the family of macromolecular objects based on purely intra-molecular bonding of single polymer chains. Among different strategies, the potential folding/collapse of individual polymer chains through pendant groups via covalent or non-covalent bonds into so-called single chain polymer nanoparticles (SCNPs) has attracted significant interest due to their potential applications in catalysis, biosensors, nanoreactors, protein mimicry, drug delivery and in nanomedicine in general [1,2]. For many reasons, SCNPs are attractive systems [1,3,4,5]. These SCNPs are unimolecular nano-objects obtained by intra-molecular cross-link of individual macromolecular chains (functionalized linear polymers called ‘precursors’). In order to prepare these SCNPs high varieties of synthetic processes have been applied to induce the intra-chain cross-linking formation [1,6,7]. Up to now, different strategies have been found in the literature where covalent cross-linking reactions, dynamic covalent chemistry, and non-covalent chemistry are the most used methodologies [1,6,7]. In general, the formation of these nanoparticles implies three steps: (i) synthesis of a precursor polymer, (ii) functionalization of the precursor polymer, and iii) folding/collapse of the functionalized precursor polymer via intra-chain interactions [5].

During the last years, similar systems to SCNPs but with a different arrangement of inter-molecular cross-links such as cyclic polymers have also emerged as potential materials. Cyclic polymers only differ from their linear analogs in their lack of chain ends (one connection). In contrast to cyclic polymers, the properties of SCNPs are dominated by both the nature and quantity of intra-molecular cross-links. The topological and/or architectural differences can have a large impact on the molecular behavior. Different chemical and physical properties, such as density [8], hydrodynamic size [8,9,10,11,12], viscosity [13,14,15,16,17], glass transition temperature [18], strength [19], and the dynamics [20], are affected by chain topology and/or architecture [21,22]. Scattering techniques present a great opportunity to study the intra-molecular cross-linking effects on SCNPs systems at a molecular level. In particular, small angle neutron scattering (SANS) and neutron spin echo (NSE) spectroscopy allow the exploration of structural and dynamical features, respectively, at the proper length scales. 

In a recent work [23,24], the structure and dynamics in melts of SCNPs based on poly(tetrahydrofuran) (PTHF) synthesized via intra-molecular azide photodecomposition process, exposed to UV irradiation (in the following denoted as ‘UV-SCNPs’), have been studied. Neutron scattering, dielectric spectroscopy, and rheological experiments were applied to explore the influence of the intra-molecular cross-linking on a bulk polymer system. While the properties at inter-molecular level—as monitored by diffraction and NSE at the first structure factor peak—were unmodified, a great impact was witnessed in the rheological behavior. In addition, a new relaxation mechanism was found associated with the internal domains originated by the intra-molecular bonds. This process was detected by dielectric relaxation and NSE at intermediate length scales, rendering the dynamics of these systems even more complex.

**Scheme 1:**
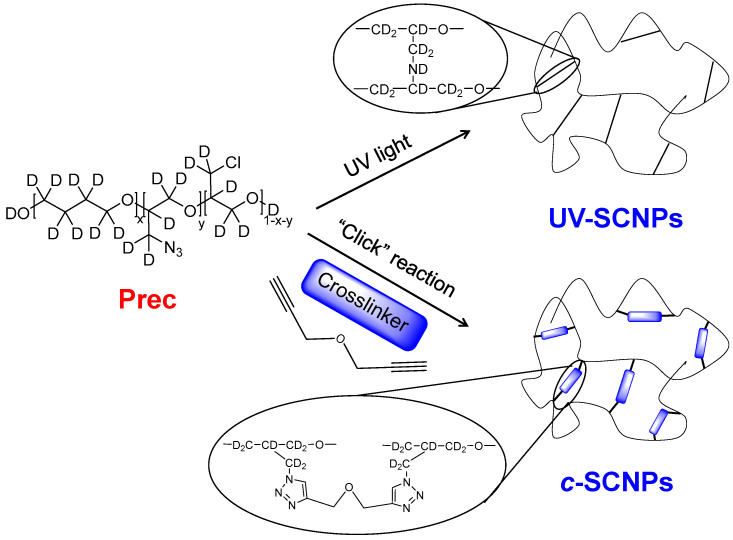
Schematic representation of the synthesis of SCNPs via intra-molecular azide photodecomposition to give “UV-SCNPs” (Ref. [25]) and via “click” chemistry using an external homobifunctional cross-linker to produce “*c*-SCNPs” (this work).

In the present work, we investigate the influence of the nature of the cross-links on the structural relaxation and rheology of bulks of SCNPs. To do this, we apply neutron scattering, thermal analysis and mechanical spectroscopy on SCNPs obtained from the same polytetrahydrofuran (PTHF)-based precursors as investigated in Refs. [23,24], but now synthesized via “click” chemistry (in the following denoted as ‘*c*-SCNPs’), as shown in Scheme 1.

Click reactions have been widely used for the synthesis of polymers with different compositions and topologies such as macrocyclic polymers [26,27], self-curable polymers [28,29], network systems [30,31], polymeric nanoparticles [32,33], etc. In particular, copper-catalyzed azide alkyne cycloaddition reactions (CuAAC, metal-catalyzed N-C click chemistry), have attracted significant attention due to their important features such as high yields, selectivity, to be applicable for a wide range of conditions and substrates, and to give stable compounds [34,35]. In the last decade, the cross-linker-induced collapse method via intra-chain CuAAC has been highly used to prepare single-chain nanoparticles of different chemical nature in a very simple manner [36,37].

To be selective to collective features in the neutron scattering experiments, we used fully deuterated samples. The direct comparison with the results on melts of their linear chains homologues evidences that the final properties (including the structural relaxation) are modified with the intra-molecular cross-links, and in a different way as in the UV-SCNPs. In particular, the presence of a oxybis(methylene)-bistriazole cross-linking moiety resulting from the “click” chemistry route (see Scheme 1) clearly influences the collective dynamics at different length scales, inducing a pronounced slowdown with respect to both, the linear precursor and the UV-SCNPs melts.

## 2. Materials and Methods 

Materials: Chemical reagents and solvents were purchased by Sigma-Aldrich, Scharlab and Eurisotop. Tris(pentafluorophenyl)borane (B(C_6_F_5_)_3_, 95%, Aldrich, Munich, Germany) was sublimed at 333 K in a cold finger condenser. Tetrahydrofuran-d_8_ (THF-d_8_, TDF, 99.5 atom% D, Eurisotop, Gif sur Yvette, France) and (±)-epichlorohydrin-d_5_ (ECH-d_5_, ECD, ≥ 98 atom% D, Aldrich, Munich, Germany) were degassed by either repetitive freeze-degas-thaw cycles. Dichloromethane (CH_2_Cl_2_, anhydrous, Aldrich, Munich, Germany) was degassed by bubbling Argon for 30 min prior to use. (+)-Sodium L-ascorbate (BioXtra, ≥ 99% (NT), Aldrich, Munich, Germany), *N,N,N′,N″,N″*-pentamethyldiethylenetriamine (PMDETA, 99%, Aldrich, Munich, Germany) CuBr was purified following the Keller and Wycoff method [38]. Propargyl ether (98%, Aldrich, Munich, Germany), *N,N*-dimethylformamide (DMF, anhydrous, ≥ 99.8%, Scharlab, Barcelona, Spain), sodium azide (NaN_3_, ≥ 99.5%, Aldrich, Munich, Germany), and methanol (MeOH, ≥ 99.9%, Scharlab, Barcelona, Spain) were used as received.

Synthesis methods: Synthesis of deuterated copolymers P(TDF-co-ECD)): The copolymers were synthesized following a similar synthetic method described by our group [25]. All reactions were carried out in bulk conditions using Schlenk flasks, under an argon atmosphere. B(C_6_F_5_)_3_ (93 mg, 0.18 mmol), THF-d_8_ (23.2 mL, 287.0 mmol), and ECH-d_5_ (5.6 mL, 71.7 mmol) were mixed in a 100 mL Schlenk flask and stirred at r.t. for 72 h. The resulting crude product was precipitated in cold MeOH and died at 333 K under reduced pressure in a vacuum oven for 24 h, getting a sticky transparent copolymer (P(TDF-*co*-ECD): 22.5 g, 68% yield, *M_w_* = 36.5 kDa, *Ð* = 1.20. The ECD content in P(TDF-*co*-ECD) was estimated to be 27 mol%.

Azidation of P(TDF-co-ECD) to obtain the deuterated precursor (Prec): 4.0 g of P(TDF-*co*-ECD) was dissolved in dried DMF (160 mL) in a round-bottom flask of 500 mL and NaN_3_ (1.7 g) was added into the solution. Then, the suspension was stirred for 24 h at 333 K. The crude product was precipitated in a cold mixture of 1:4 H_2_O/MeOH mixture and dried at 50 °C under reduced pressure in a vacuum oven for 24 h to obtain the azide-containing deuterated precursor (Prec: 7.2 g, 75% yield).

**Figure 1:**
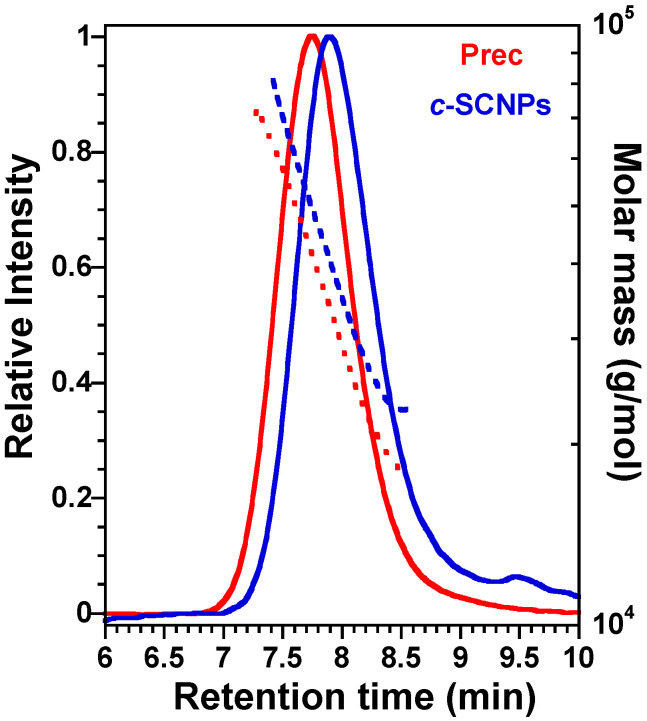
SEC chromatograms of Prec and *c*-SCNPs showing the shift of the SEC retention peak and molar mass of Prec upon chain compaction via “click” chemistry to produce *c*-SCNPs.

Synthesis of click-deuterated single-chain nanoparticles (c-SCNPs): SCNPs were synthesized via copper-catalyzed azide alkyne cycloaddition. CuBr (0.86 g, 6 mmol), (+)-Sodium L-ascorbate (1.190 g, 6 mmol) and PMDETA (1.26 mL, 6 mmol) were added into a round-bottom flask and purged with argon for 15 min. Then, a solution of 150 mg of precursor (Prec) in dried CH_2_Cl_2_ (150 mL, 1 mg mL^−1^) was added into the mixture under an argon atmosphere. A solution of propargyl ether (7 µL, 0.072 mmol) in 7 mL of CH_2_Cl_2_ was injected to the mixture with a syringe pump at 7 mL h^−1^ under argon. After the complete addition of the cross-linker solution, the reaction was stirred at room temperature for 24 h. The cooper catalyst was removed by extraction with a saturated solution of NH_4_Cl (4 × 100 mL). The organic phase was dried with MgSO_4_, filtrated and evaporated at reduced pressure to obtain *c*-SCNPs as greenish viscous liquids (126 mg, 80% yield). To confirm that the SCNPs are formed, SEC analysis was carried out and was compared with the precursor’s one (Figure 1).

Sample preparation: A solution containing either the precursor or the SCNPs and 2 mL of CH_2_Cl_2_ was stirred until completed dissolution. Then, the solution was drop-casted onto the aluminum flat holders and the solvent was slowly evaporated in the fume hood. Finally, the samples were well-dried in an oven under vacuum at 343 K for 24 h.

Size-exclusion chromatography (SEC): SEC measurements were performed at 303 K on an Agilent 1200 system equipped with PLgel 5 μm Guard and PLgel 5 μm MIXED-C columns (Santa Clara, California, USA), and triple detection: a differential refractive index (RI) detector (Optilab Rex, Wyatt), a multi-angle laser light scattering (MALLS) detector (MiniDawn Treos, Wyatt Technology Corporation, Santa Barbara, CA, USA), and a viscosimetric (VIS) detector (ViscoStar-II, Wyatt Technology Corporation, Santa Barbara, CA, USA). Data analysis was performed with ASTRA Software (version 6.1) from Wyatt Technology Corporation, Santa Barbara, CA, USA. THF was used as eluent at a flow rate of 1 mL/min. A value of dn/dc = 0.062 was used for precursors and SCNPs.

Thermal analysis: A differential scanning calorimeter (DSC) TA instrument Q2000 (TA Instruments, New Castle, DE, USA) was used to measure the calorimetric glass transition temperature (*T_g_*) of the samples. All the experiments were performed under ultrapure nitrogen flow, and samples of 5 mg were used. First, non-isothermal scans were performed, where the samples were heated up until 350 K, and kept here for 3 min, where the temperature is high enough to erase any previous thermal history. Then a cooling scan at 20 K/min was recorded to 170 K, followed by a subsequent heating scan also at 20 K/min from this temperature to 350 K. The *T_g_* values were extracted from the onset value of the jump in heat capacity.

Mechanical analysis: Rheological experiments in the linear regime in the frequency range ≈ 0.03–16 Hz were performed by using an ARES rheometer (TA Instruments, New Castle, DE, USA ) with a parallel plate 8 mm diameter geometry with a gap distance of ≈0.4–0.5 mm. The strain amplitude was 2% to ensure a linear regime. Measurements were carried out under a N_2_ atmosphere, and the temperature was set by an LN2 controller. The samples were measured at temperatures 213 ≤ *T* ≤ 313 K.

Structural analysis: The short-range order of the systems was studied by wide angle X-ray scattering (WAXS) on a Bruker D8 Advance diffractometer (Bruker, Bremen, Germany) working in parallel beam geometry with Cu K_α_ transition photons of wavelength λ = 1.54 Å. The experiments were performed at room temperature in reflection mode (θ–2θ configuration), varying the scattering angle 2θ from 10° to 30° with steps of 0.05°. The scattered intensities are shown as a function of momentum transfer *Q*, *Q* = 4π*λ*^−1^ sin *θ*. The low *Q*-region was explored by means of the D22 SANS instrument at the Institute Laue Langevin (ILL) in Grenoble [39]. Using *λ* = 6 Å, the scattered intensity was recorded with sample-detector distances of 17.6, 5.6 and 2 m to cover a *Q*-range from 0.003 to 0.5 Å^−1^. Four different temperatures were explored: 280, 300, 320 and 360 K.

Dynamic structure factor: The coherent intermediate scattering function or dynamic structure factor of deuterated SCNPs and their precursors in bulk were studied with the NSE spectrometer IN11c also at the ILL [39]. The multidetector at IN11c covers an angular range of 30° in the horizontal plane. It was placed at 20°, 50°, and 80° scattering angle for its central detector. With an incident wavelength of 5.5 Å, a *Q*-range of 0.15 ≤ *Q* ≤ 1.64 Å^−1^ and a time range from 5 ps to 0.7 ns were explored. The same temperatures as in SANS were investigated.

## 3. Results

Before presenting the results on the bulk systems, we show in Figure 1 the successful formation of *c*-SCNPs from Prec via a “click” chemistry reaction prior to melt sample preparation. The figure shows how the *c*-SCNPs obtained have longer retention time at the SEC peak maximum compared to their precursor. At the same time, the *c*-SCNPs obtained were unimolecular, as molar mass distribution remained constant.

**Figure 2:**
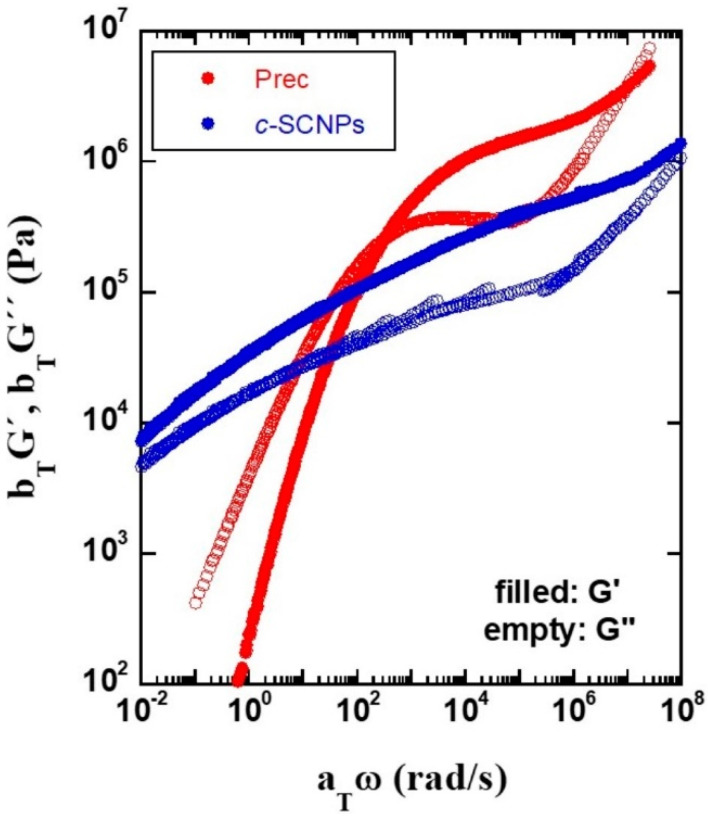
Rheological master curves (reference temperature: 293 K) for precursor and *c*-SCNPs.

The *T_g_* value of the precursor melt determined by DSC was 202 K, while in the *c*-SCNPs melt this value increases by 5 K, up to 207 K. The mechanical response of the *c*-SCNPs with respect to its linear precursor was studied by rheological experiments. The master curves obtained for the real and imaginary parts of the shear modulus are presented in Figure 2. They were obtained applying the time-temperature superposition (TTS) principle to isothermal curves of *G′*(*ω*) and *G″*(*ω*) with a reference temperature of *T_ref_* = 293 K. The Prec sample exhibits the typical long linear chain behavior. At intermediate high frequencies, the elastic modulus *G′*(*ω*) is above *G″*(*ω*), reflecting a solid-like behavior and the curves cross at two points: at high frequencies (about 10^7^ rad/s), where the onset of entanglement effects on the mechanical response is reflected, and at low frequencies (3 × 10^2^ rad/s) where chains disentangle. For the *c*-SCNPs sample, the plateau in the shear modulus smears out and no disentanglement point can be identified in the experimental window accessed. This indicates that, at the temperatures and frequencies probed, the system doesn´t flow, reminding the behavior of a gel. We also note that in the case of the *c*-SCNPs the obtained absolute values for the moduli are rather low. This effect could be due to uncertainties in the geometry of the sample.

**Figure 3:**
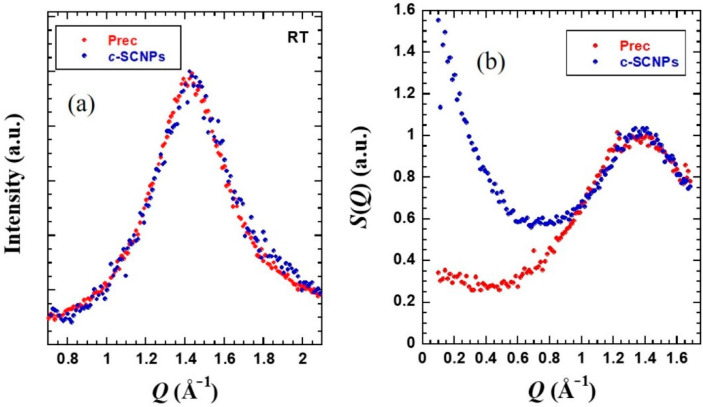
(**a**) WAXS diffraction patterns determined at room temperature for precursor and *c*-SCNPs. (**b**) Static structure factor obtained by IN11c on precursor and *c*-SCNPs at 320 K. In both cases data have been scaled to match at the maximum.

WAXS experiments provided information on the short-range order of the samples. Figure 3a shows the obtained results. One main peak is observed in the *Q*-range between 1.0 and 2.0 Å^−1^ centered around 1.4 Å^−1^. This peak can be understood as due to inter-main chain correlations with associated average distances of about *d*_chain_ = 2π/*Q*_max_ ≈ 4.4 Å [40]. This peak is not visibly disturbed by internal cross-links.

While X-rays are sensitive to the electronic probability density, neutron diffraction on fully deuterated samples addresses the total structure factor *S(Q),* where all nuclei pair correlations are equally weighted. IN11c in diffraction mode delivers this function (Figure 3b). The peak position here is also unaffected by the presence of internal cross-links. The *Q*-range accessed by this instrument extends toward lower *Q*-values than the WAXS measurements. In the low-Q range explored, the coherent scattering of the *c*-SCNPs exhibits a marked increase with decreasing *Q*-value that is not present in the precursor melt. The region of *Q*-values where this effect is noticed is already located in the regime where we explore the so-called intermediate length scales (ILS)–the range of length scales larger than the inter-molecular distances but not yet in the hydrodynamic regime. 

**Figure 4:**
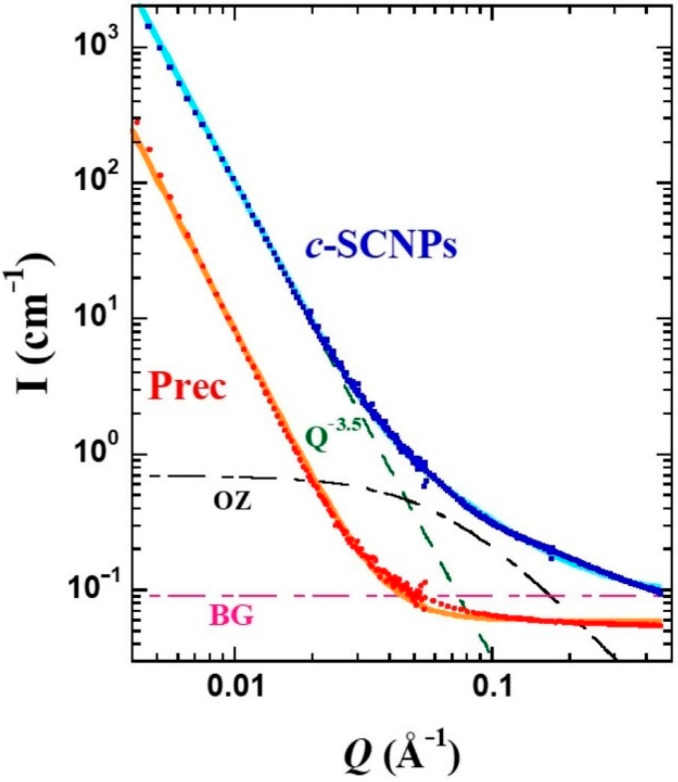
Differential cross section of Prec (red circles) and *c*-SCNPs (blue squares). The orange solid line is a description of the data for Prec at intermediate length scales, consisting of a ∝ Q^−x^ law with x = 3.5 and a constant background. The blue solid line describes the *c*-SCNPs data, including an Ornstein-Zernike contribution (black dashed-dotted line), a ∝ Q^−x^ law with x = 3.5 (green dashed line) and a constant background (pink dashed-dotted line).

A deeper scrutiny of this feature was possible by applying SANS. Figure 4 displays the differential cross sections for both samples. To describe this region of *S*(*Q*) in the precursor, a constant function has been considered, as expected for a homogeneous system at large length scales with respect to the inter-molecular distances. This describes well the behavior up to ≈0.08 Å^−1^, value below which a strong increase of the intensity is observed. Such a low-*Q* contribution can be accounted for by a law ∝ *Q*^−x^, with x = 3.5. This additional scattering is usually found in fully deuterated polymer melts and could be attributed to the presence of microbubbles. The combination of these two contributions well describes the precursor results. As observed by IN11c, in the *Q*-region around 0.1 Å^−1^ the *c*-SCNPs show an excess of the scattered intensity compared to the precursor sample. This difference can be considered by an Ornstein–Zernicke (OZ) expression:

(1) $$I_{OZ\;}\left(Q\right)\;=\;\frac{I_{OZ}\;\left(0\right)}{1\;+\;{\left(\xi Q\right)}^{2}}$$

where *I_OZ_* (0) is the amplitude or *Q* → 0 value of this function and *ξ* is the correlation length. To give account for the intensity scattered by the *c*-SCNPs in the whole range accessed by SANS, this contribution was added to the power law and constant background contributions as considered for the Prec sample. As can be seen in Figure 4, the resulting total function (solid line) describes well the experimental results. There, the three separate contributions are also displayed. The value of x in the power law found was the same as for Prec, x = 3.5. Regarding the OZ contribution, a value of 0.7 cm^−1^ was obtained for the amplitude, and *ξ* ≈ 15 Å for the characteristic length. The SANS results were insensitive to temperature, in the range investigated. 

**Figure 5:**
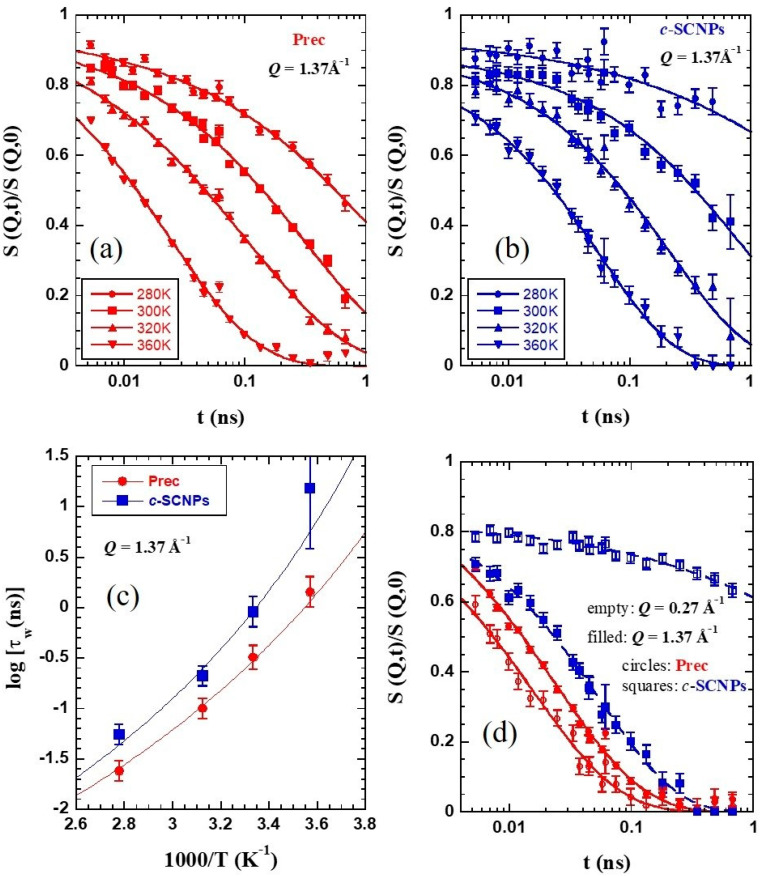
Normalized dynamic structure factor at *Q* ≈ *Q_max_* of (**a**) Prec and (**b**) *c*-SCNPs samples at different temperatures, (**c**) temperature dependence of the collective characteristic times from NSE at *Q* = 1.37 Å^−1^ and (**d**) a comparative between Prec and SCNPs samples at *Q* ≈ *Q_max_* and a representative *Q* value in the ILS region at 360 K. Lines are fits of stretched exponentials functions for the dynamic structure factor and of the Vogel-Fulcher equation for the characteristic times.

Neutron Spin Echo experiments on the deuterated samples reveal collective dynamics. From the above structural results (WAXS and the polarization data from IN11c), we know that the structure factor presents an amorphous halo centered at ≈ 1.4 Å^−1^. At this value (*Q_max_*) NSE directly monitors the temporal evolution of inter-chain correlations. Figure 5a,b show the NSE results on the normalized dynamic structure factor *S*(*Q*,*t*)/*S*(*Q*,*0*) at a *Q*-value close to *Q_max_* and the different temperatures investigated on Prec and *c*-SCNPs, respectively. At first sight, we can observe a clear slowing down of the dynamic structure factor when the sample contains internal cross-links in the whole T-range studied. The curves can be well described by Kohlrausch–Williams–Watts (KWW) functions

(2) $$\frac{S\left(Q,t\right)}{S\left(Q,0\right)}\;\propto \exp\left[-{\left(\frac{t}{\tau_{w}}\right)}^{\beta }\right]$$

where *β* is the stretching exponent and *τ_w_* the characteristic time. The fits of Equation (2) to the experimental data show a variation of *β* with temperature. In the temperature range investigated, it increases from 0.47 to 0.6 for Prec and from 0.39 to 0.69 for *c*-SCNPs. In Figure 5c the temperature dependence of the collective characteristic time at this *Q*-value—the usually called structural relaxation time—is plotted for both samples. As expected from the simple inspection of the NSE curves, the structural times are longer for the *c*-SCNPs than for their precursor counterparts for all T-values studied, in accordance with the higher value of the glass-transition temperature *T_g_* observed by DSC. The precursor times can be well described by the Vogel-Fulcher (VF) function

(3) $$\tau \;\left(T\right)\;=\;\tau_{\infty }\exp\left[\frac{B}{T\;-\;T_{0}}\right]$$

with the values of the Vogel temperature *T_0_* and the energetic term *B* as determined by dielectric spectroscopy for the α-relaxation [23], *T_0_* = 161.4 K and *B* = 1125 K. The prefactor used is *τ_∞_* = 9 × 10^−5^ ns. The *c*-SCNPs results can be reasonably described by keeping the same values of *τ_∞_* and *B*, and assuming a shift in the Vogel temperature. This is found to be of 17 K, i.e., larger than the shift observed for the glass-transition temperature.

**Figure 6:**
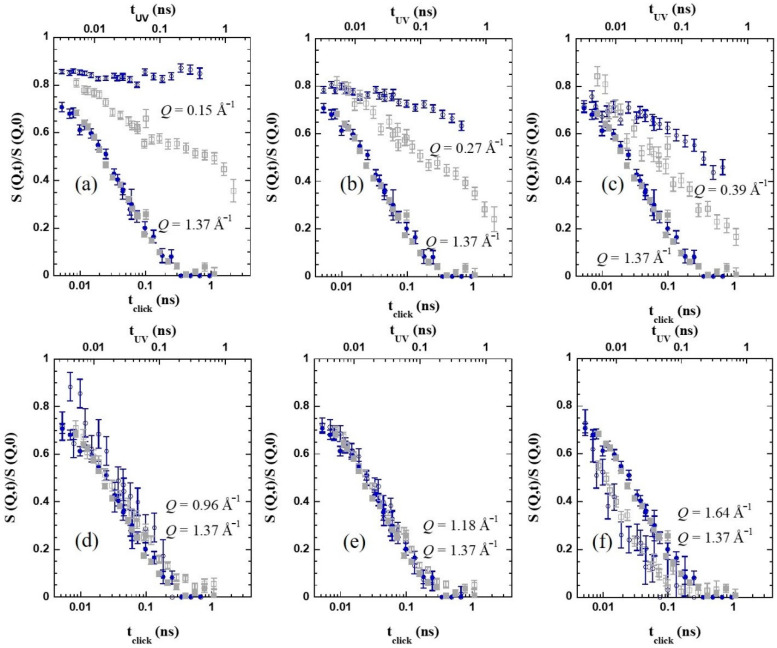
Normalized dynamic structure factor at *Q* ≈ *Q_max_* (filled symbols) and a different *Q* values (empty symbols), (**a**) *Q* = 0.15 Å^−1^, (**b**) *Q* = 0.27 Å^−1^, (**c**) *Q* = 0.39 Å^−1^, (**d**) *Q* = 0.96 Å^−1^, (**e**) *Q* = 1.18 Å^−1^, and (**f**) *Q* = 1.64 Å^−1^ at 360 K for SCNPs samples synthesized via “click” chemistry (*c*-SCNPs, circles) and via intra-molecular azide photodecomposition (UV-SCNPs, squares). They are plotted vs the corresponding Fourier time scale (lower x-axis for the *c*-SCNPs, upper x-axis for the UV-SCNPs).

The difference in the dynamical behavior is even more evident when the comparison of the NSE results is performed at *Q* values below *Q_max_*, in the intermediate length scales (ILS) regime. This is illustrated in Figure 5d for a Q-value of 0.27 Å^−1^. While for the linear chains the decay of the density correlation function is faster than at *Q_max_*, for the *c*-SCNPs the dynamic structure factor displays a much more pronounced stretching and decays at much longer times (more than one decade) than the inter-molecular correlations. The blue circles in Figure 6a–c show that this effect is found over a large Q-range below the structure factor peak. In particular, collective motions appear to be frozen in the accessed dynamic window for *Q*=0.15 Å^−1^. Thus, the presence of cross-links strongly hampers the relaxation of collective motions of the chains in this regime.

## 4. Discussion

Except for the average inter-molecular distances (position of the amorphous halo), all the properties investigated in this work for the bulk of SCNPs prepared through “click” chemistry (*c*-SCNPs) clearly differ from those of the melt of corresponding linear precursor chains. The calorimetric glass transition occurs at 5 K higher temperature; accordingly, longer characteristic times are observed for the dynamic structure factor at the inter-molecular level directly revealing the structural relaxation. Unfortunately, the limited temperature window accessed and the uncertainties at the lowest temperature investigated do not allow a precise determination of the VF-parameters for the samples only from the NSE results. However, it is clear that the data of the *c*-SCNPs demand an increase in the Vogel temperature with respect to that of the precursor, and would also be compatible with changes in the other parameters. Thus, even though the short-range order (average distances between adjacent chains) is not appreciably disturbed by the cross-links, the structural relaxation is significantly slowed down, in particular with decreasing temperature. The main impact is nevertheless observed at intermediate length scales, where collective features—both static and dynamic—are profoundly affected by cross-links. Last, they clearly impact the rheological behavior of the sample.

To discuss the origin of these effects on the different properties, it is very helpful to consider in a comparative way our previous works on bulk of SCNPs obtained via intra-molecular azide photodecomposition process (UV-SCNPs) from similar precursors [23,24]. Starting with the structural features, in that case we also observed insensitivity of the inter-molecular distances and enhanced scattered intensity at intermediate length scales. This excess of scattering was also described in terms of an OZ function, yielding a value of 0.5 cm^−1^ for the amplitude and 9 Å for the correlation length of this contribution. Thus, the excess of density fluctuations induced by the cross-links is more pronounced –according to the larger amplitude—and the associated characteristic length is larger when employing the “click” chemistry method. Such an excess reveals structural heterogeneities in the melt. The origin of these heterogeneities can be attributed to the internal compartmentation of the SCNPs into ‘domains’ [41]. According to molecular dynamics simulations [41], we expect the presence of chain portions forming loop-like structures within the chains (‘domains’), which sizes would be broadly distributed. Our results would suggest the presence of larger domains in average in the case of the “click” synthesis route involving the generation of relatively large oxybis(methylene)-bistriazole cross-linking moieties. We note that the size of these moieties is already about 1 nm [42], versus the atomic bond size (of the order of 1–2 Å) of the links in the UV-SCNPs.

Moving to the dynamical features, we first consider the impact of cross-links on the structural relaxation as directly observed by NSE at the first structure factor peak *Q*_max_. Contrarily to the bulk of *c*-SCNPs here investigated, that consisting of UV-SCNPs showed exactly the same behavior as the linear precursor counterparts for this process. No shift in the T_g_-value was either observed. This implies that the structural dynamics of UV-SCNPs is faster than that of the present *c*-SCNPs. Figure 6 compares the NSE results for both systems at the highest temperature investigated. The filled symbols correspond to the *Q*-value close to *Q*_max_. The data from the two samples nearly coincide if a relative shift factor in the time scales of 1.6 is applied for this particular temperature; this would be the ratio between the structural times of the two samples, τ_click_ = 1.6 τ_UV_, at 360 K. Thus, we can infer that the nature of the cross-linker does strongly affect the dynamics at inter-molecular level. The presence of a relatively large oxybis(methylene)-bistriazole cross-linking moieties significantly slows down the decay of the inter-molecular correlations, with respect to the case when covalent bonds directly linking chain monomers are created (see Scheme 1). This difference increases with decreasing temperature, according to the thermal behavior observed for the structural relaxation time (Figure 5b).

Regarding the impact of cross-links at other length scales, the different panels in Figure 6 show the NSE results for the two SCNPs samples at various *Q*-values, always compared with the data of *Q =* 1.37 Å^−1^ as reference, at 360 K. The above commented shift in the time applied in the figures allows appreciating how different is for the two samples the relative behavior at a given *Q*-value with respect to the structural relaxation at the first peak. We can see that, around the maximum of the peak (explored *Q*-values above approx. 1 Å^−1^) the behavior of the dynamic structure factor is identical, within the experimental uncertainties, for both bulk systems. If we move toward lower *Q*-values, however, the behavior starts to differ. Entering the ILS region, as shown in Figure 6a–c for *Q =* 0.15, 0.27, and 0.39 Å^−1^, respectively, the *c*-SCNPs present a clear slowing down of the dynamic structure factor, compared with the UV-SCNPs. We note that the latter also exhibit a clear effect of internal cross-links in this regime, consisting of an enhanced stretching and a slowing down with respect to the linear precursor chains. However, in this case, the impact is less pronounced than for the *c*-SCNPs bulk. For both melts, these effects could be attributed to the emergence of new constraints related to the internal domain topology. This internal structure would lead certain correlations to persist over longer times. Close to cross-links, the conformation of the chain and the packing would be different from the average in the melt. These properties would also change from one domain to another. Segments chemically connected by the cross-links would feel an extra friction to relax. This would provoke the retardation of the decay of the density fluctuations at the corresponding length scales (as deduced from the SANS results, in the nanometric range). As it was proposed for the UV-SCNPs, the complete decay of the dynamic structure factor would only be reached through additional mechanisms for relaxation not present in the linear precursor chains. Due to the topological complexity induced by the cross-linking synthesis, a hierarchy of relaxation mechanisms would be in fact expected to occur in order to reach the full relaxation of the SCNPs in the melt. We mention that dielectric spectroscopy studies on the UV-SCNPs bulk pointed to the emergence of an additional dynamic contribution to dipolar relaxation intermediate between the α-process and the Rouse regime that would be related to the relaxation mechanism involving the internal domains in the SCNPs [23]. Apparently, these effects are more marked in the *c*-SCNPs. We note again the bigger size of the involved cross-links in this case, that could be behind this observation. The scrutiny of the rheological results, as argued below, can also provide a plausible explanation of this finding.

As mentioned above, the absence of flow in the dynamic window explored by the rheological experiments suggests a kind of gel-like behavior for the *c*-SCNPs. This behavior is a priori not expected in the cases where there is exclusively intra-chain bonding as it has been found, for instance, in ring polymer melts that have a smaller disentanglement time and lower viscosity than their linear chain counterparts [43]. Similarly, our work on UV-SCNP melts showed a much lower relaxation time compared to its linear precursor [23], suggesting in fact a suppression of entanglements with respect to the linear case. A gel-like behavior as that here observed for the *c*-SCNPs would be expected when inter-chain bonding occurs, i.e., in a 3D network. However, SEC results clearly show that the polymer molecular weight does not increase significantly upon cross-linking reaction (Figure 1) confirming that the formed bonds are exclusively intra-molecular after synthesis. On the other hand, in principle, the type of internal cross-linking is not expected to affect the long-time rheological behavior. We may attribute the long time rheological behavior to the effect of residual copper ions from the catalytic reaction. These ions, together with the possible presence of a remaining fraction of pending (partially unreacted) cross-linkers, could give rise to the formation of some inter-molecular crosslinks among different SCNPs when they are condensed in a bulk system. Even if the inter-molecular bridges are extremely scarce, they can lead to a huge impact in the mechanical properties of the melt. These additional constraints to flow would also explain the slowing down of the dynamic structure factor of the present *c*-SCNPs with respect to the UV-SCNPs at intermediate length scales above discussed. We also note that the *c*-SCNPs system could not be dissolved and diluted after the experiments, to perform a posteriori SEC analysis. This observation also confirms the formation of a gel-like structure after the condensation of these nanoparticles in a bulk material with reminiscences of copper ions [44,45]. Finally, we also note that the overlap of the *G’’(w)* in the master curve is clearly worse than in the precursor melt. This could be attributed to a complex underlying superposition of relaxation processes with different activation energies, in agreement with the scenario here proposed to explain the particularly pronounced slowing down of the dynamic structure factor in the melt of *c*-SCNPs, especially at intermediate length scales.

## 5. Conclusions

With a combined methodology that includes thermal analysis, rheological measurements, diffraction, and quasielastic neutron scattering, we have scrutinized the structure and dynamics of melts of polymers before and after applying a synthesis route that produces intra-molecular crosslinks within the individual chains (leading to SCNPs formation). The route followed in this work (via “click” chemistry) involves the generation of relatively large oxybis(methylene)-bistriazole cross-linking moieties. The comparison with results on an analogous system synthesized via intra-molecular azide photodecomposition has allowed us to elucidate the properties that universally arise in bulks of intra-molecularly bonded chains and those that depend on the kind of cross-link involved. In view of the results, we can state that, generically, internal cross-links do not influence the average inter-molecular distances in the melt, while they have a profound impact at intermediate length scales. This concerns both the structure—through the emergence of heterogeneities at the nanometric scale, and the dynamics—leading to a more complex relaxation behavior including processes that allow relaxation of the internal domains.

The influence of the nature of the internal bonds is seen in the structural relaxation. This process is slowed down if somewhat bulky cross-linking agents are involved in the bonds between the functionalized monomers, while no effect is found if these monomers are directly connected with covalent bonds.

We have also observed that a residual amount of copper ions from the catalytic reaction can lead to further reaction in the bulk material and produce inter-molecular bonding. Even if the amount of such links is tiny, their impact in the rheological behavior is huge. In this case, the system behaves like a gel. This effect is antagonistic to the entanglement suppression observed for the case of the SCNPs obtained through intra-molecular azide photodecomposition (UV-SCNPs), where only intra-molecular cross-links were produced. The presence of inter-molecular cross-links could also be responsible for the additional hindering of the decay of density fluctuations observed by NSE for the *c*-SCNPs bulk at intermediate length scales.

Finally, the larger correlation length and amplitude of the extra-contribution to the SANS patterns in the case of *c*-SCNPs with respect to the UV-SCNPs could be either due to the different nature of the induced cross-links or to the presence of inter-molecular bridges. Discerning between these two scenarios will be subject of future work. Another important ingredient, the internal cross-linking density, will also be systematically investigated to have a full picture.
